# Socioeconomic inequalities in contraceptive use among female adolescents in south Asian countries: a decomposition analysis

**DOI:** 10.1186/s12905-022-01736-8

**Published:** 2022-05-10

**Authors:** Himani Sharma, Shri Kant Singh

**Affiliations:** 1 Department of Mathematical Demography and Statistics, International Institute for Population Studies, Mumbai, India; 2 Department of Mathematical Demography and Statistics, International Institute for Population Studies, Mumbai, India

**Keywords:** South Asia, Inequality, Contraceptive, Adolescents, Contraception

## Abstract

**Background:**

Contraceptive knowledge and use has been an emerging topic of interest in adolescents in Asia. This study quantified the contribution of the socioeconomic determinants of inequality in contraceptive use among currently married female adolescents (15–24) in four south Asian countries: India, Bangladesh, Nepal and Pakistan.

**Data and methods:**

The data of Demographic Health Survey (DHS) for four South Asian countries, i.e. India (NFHS 2015–16), Nepal (DHS 2016), Bangladesh (DHS 2014) and Pakistan (DHS 2012–2013) has been used for examining the contraceptive use and inherent socioeconomic inequality. After employing logistic regression, concentration curves based on decomposition analysis have been made to analyse the socioeconomic inequality.

**Results:**

The results reveal that the use of contraception among female adolescents remains low and factors like education, employment, having one or more children, media exposure were positively associated with it. In terms of socioeconomic inequality, a significant amount of variation has been observed across the countries. In India, poor economic status (95.23%), illiteracy (51.29%) and rural residence (23.06%) contributed maximum in explaining the socioeconomic inequality in contraceptive use among female adolescents. For Bangladesh, the largest contributors to inequalities were rural residence (260%), illiteracy (146.67%) while birth order 3 + (− 173.33%) contributed negatively. Illiteracy (50%), poor economic status (47.83%) and rural residence (16.30%) contributed maximum to the inequalities in contraceptive use in Pakistan while birth order 3 + (− 9.78%) contributed negatively. In Nepal, the important operators of inequalities were unemployment (105.26%), birth order 3 + (52.63%) and poor economic status (47.37%), while rural residence contributed negatively (− 63.16%) to inequalities in contraceptive use.

**Conclusions:**

Using a cross country perspective, this study presents an socioeconomic inequality analysis in contraceptive use and the important factors involved in the same. Since the factors contributing to inequalities in contraceptive use vary across countries, there is a need to imply country-specific initiatives which will look after the special needs of this age-group.

## Background

Adolescence is considered as a significant stage of an individual’s life. It encompasses a lot of biological and psychosocial changes that affect every aspect of adolescents’ lives. It is for these changes that adolescence becomes a unique life course experience. In addition, it is also referred as an imperative period which puts forward the foundations of good health and wellbeing in the adulthood [1]. However, sexual maturation or puberty go hand in hand with the physical growth of the adolescents leading to intimate relationships at an early age in case of inappropriate guidance and incomplete information. It thus, becomes important to promote sexual literacy, wherein sex education can contribute to positive psychosocial development and well-being throughout adolescence and adulthood [2].

Contraceptive knowledge and use has been an emerging topic of interest in adolescents in Asia. Of the estimated 1.2 billion adolescents in the world today, nearly half live in Asia, and nearly one in four (282 million) live in South Asia.. Within this region, Bangladesh and Pakistan have the greatest proportion of adolescents, while India has the greatest absolute number. There has been much ambiguity in defining the age group of adolescents throughout the world. The World Health Organization (WHO) and United Nations define adolescents as the age group 10–19. However, Demographic Health Survey (DHS) treats the age group of 15–24 as adolescents. The family planning programs worldwide are focused towards addressing the needs of adolescent’s women as they enter into sexual union early and are exposed to the risk of pregnancy. Although many teenagers know about family planning, the levels of contraceptive knowledge among adolescents lags behind when compared to older age groups; adolescents are unlikely to use a contraceptive the first time they have sex and are more likely than older women to experience a contraceptive failure [3]. South Asia and Asia have many similarities and differences in common; evidences of which is provided in a study by [4] stating that countries in South Asia (except Sri Lanka) are characterized by early marriage, and early childbearing within marriage is prevalent in both regions. Thus, married adolescent women in Asia constitute a uniquely vulnerable population with special needs. Although they have many common grounds for research, they differ in many ways depending on their culture and society.

A number of literatures are available on Asian adolescents focusing upon the issue of low contraceptive use and high unmet need among them. The evidence of low contraceptive prevalence among South Asian adolescents is plenty; although awareness of contraception is almost universal among married adolescents, knowledge of specific methods and sources of supplies is limited [4]. Adolescent women are less likely to be knowledgeable about family planning than are adult women, and they are much less likely than are adult women to be practicing contraception; in addition, compared with adult women, contraceptive use among adolescents is more likely to result in an undesirable outcome-an unplanned pregnancy or an unmet need for a method [5]. Many studies across South Asia have been conducted pertaining to this issue. One such study based in Nepal revealed that many adolescents in Nepal lack the power and skill to use contraceptives, especially young women who must negotiate the use of condoms with a male partner [2]. In 1995, Bangladesh Rural Advancement Committee set up an initiative of providing sexual and reproductive education among adolescents’ trough local schools and community libraries [6]. Despite this, the percentage of adolescents not using any contraceptive is high in Bangladesh. The study about the use of contraceptives among adolescents reveals an issue of key importance to this particular group, namely, that adolescent girls may know about contraceptives but do not necessarily use them [7]. A study in Pakistan shows an important cause of large gap between intended and actual births is the low contraceptive prevalence rate in the country, the reasons for which have been widely discussed in a number of studies, i.e., the socio- cultural values and gender inequality issues [8]. The family planning programmes have a major role to play in contraceptive use and prevalence among adolescents. Clients who have more contacts with the family planning program use contraceptives more consistently than those with fewer contacts [9]. Most of the earlier research has been done on the prevalence, levels and trends of contraceptive use using cross-sectional data of individual countries or south Asian countries [10, 11]. No previous study to our knowledge has investigated the determinants and socioeconomic inequalities in contraceptive use using cross country analysis. Moreover, there exists a dearth of studies when it comes to adolescent contraceptive behavior in the south Asian region. This study is also unique as it mainly focuses on the adolescent population. Therefore, the main aim of the present study was to (a) identify the prevalence and determinants of contraceptive use among adolescents aged 15–24 in four south Asian countries; (b) examine the association between sociodemographic and behavioral characteristics and contraceptive use and (c) decompose the socioeconomic inequalities in contraceptive use among adolescents aged 15–24 in India, Bangladesh, Nepal and Pakistan.

### Methods

#### Study design

The present study is a cross-sectional study based on secondary data from four South Asian countries.

#### Setting

The nationally representative Demographic Health Survey (DHS) for four South Asian countries, including, India (National Family Health Survey (NFHS) 2015–16), Nepal (DHS 2016), Bangladesh (DHS 2014) and Pakistan (DHS 2012–2013) was used for the present study.

#### Participants

The Woman’s Questionnaire of DHS collected information from all eligible women aged 15–49, who were asked questions on varied topics like background characteristics, reproduction, family planning, maternal and child health, breastfeeding, and nutrition, marriage and sexual activity, fertility preferences, husband’s background and woman’s work and other health issues. The survey provides data on a different dimensions of women’s sexual and reproductive health for the reproductive age group 15–24 across states and districts of India, Bangladesh, Nepal and Pakistan. In the case of India, with a response rate of 98 per cent, a total of 601,509 households were successfully interviewed.

### Variables

#### Outcome variable

*Modern contraceptive use*: Percentage of modern method of contraception used by currently married adolescents aged 15–24. Modern methods include male and female sterilization, injectables, intrauterine devices (IUDs/PPIUDs), contraceptive pills, implants, female and male condoms, diaphragm, foam/jelly, the standard days method, the lactational amenorrhoea method, and emergency contraception.

#### Explanatory variables

The explanatory factors included many variables reflecting socio-economic as well as some behavioural characteristics. The variables were used on the basis of many earlier and recent studies. For a better comparative analysis, the variables were kept the same for all four south Asian countries. The variables included in the analysis were age, place of residence, education, religion, wealth index, working status, age at first sex, number of living children, husband’s education, media exposure, visits by FP worker. In the DHS surveys, households are given scores based on the number and kinds of consumer goods they own, ranging from a television to a bicycle or car, and housing characteristics such as the source of drinking water, toilet facilities, and flooring materials. These scores are derived using principal component analysis. National wealth quintiles are compiled by assigning the household score to each usual (de jure) household member, ranking each person in the household population by their score, and then dividing the distribution into five equal categories, each with 20 per cent of the population [12].

*Data source/measurement*: The nationally representative Demographic Health Survey (DHS) has been used for the present study. The data from four South Asian countries, i.e. India (NFHS 2015–16), Nepal (DHS 2016), Bangladesh (DHS 2014) and Pakistan (DHS 2012–2013) has been used for examining the contraceptive use and inherent socioeconomic inequality. These countries were selected because of the highest amount of adolescent population in these countries. DHS follows a uniform system of sampling across all the countries. Therefore, the data being representative at each level of stratification (region, district, state or nation). The NFHS-4 sample is a stratified two-stage sample. The 2011 census served as the sampling frame for the selection of PSUs. PSUs were villages in rural areas and Census Enumeration Blocks (CEBs) in urban areas. In every selected rural and urban PSU, a complete household mapping and listing operation were conducted prior to the main survey [12].

*Study size*: The analysis in the present study is limited to currently married female adolescents of the 15–24 age group. The final sample size for the study was 94,034 for India, 5074 for Bangladesh, 2569 for Pakistan and 2474 for Nepal.

### Statistical Methods

#### Bivariate analysis and logistic regression

The analysis starts with the bivariate analysis in order to analyze the contraceptive use among ever married adolescents in South Asian countries by their socio-economic characteristics. It has been followed by logistic regression which helps us to assess the effects of the socio-economic characteristics on contraceptive use among adolescents.

#### Concentration and decomposition analysis

Inequality measures have been the most convenient way of analyzing the inequalities in health and related areas. The common examples are concentration index, poor rich ratios, regression analysis etc. these methods can only look into the differences and inequalities in health.

The *concentration index* and related *concentration curve* provide a means of quantifying the degree of income-related inequality in a specific health variable. The concentration index is defined with reference to the concentration curve (q.v.), which graphs on the x-axis the cumulative percentage of the sample, ranked by living standards, beginning with the poorest, and on the *y*-axis the cumulative percentage of the health variable corresponding to each cumulative percentage of the distribution of the living standard variable. The concentration index (*C*) can be computed very simply by making use of the “convenient covariance”:

$$C=\frac{2}{\mu }{COV}_{W }({y}_{i}{R}_{i})$$

where ${y}_{i}$ is the health variable whose inequality is being measured, μ is its mean, ${R}_{i}$ is the *i*th individual’s fractional rank in the socioeconomic distribution (e.g. the person’s rank in the income distribution), and cov (.,.) is the covariance. Where the data are weighted, a weighted covariance needs to be computed, and a weighted fractional rank needs to be generated [13].

A concentration curve that lies above the line of equity represents a situation where poor maternal health care utilization is more concentrated among the ‘disadvantaged’ population [14]. The amount of contribution of each factor influencing inequality cannot be obtained by these measures. To overcome this problem, Wagstaff (2003) gave an inequality decomposition model for assessing inequalities. All the statistical analyses have been done using the Stata software (version 14), whereas the concentration graphs have been computed using Microsoft excel.

### Results

#### Profile of the respondents

Table 1 shows the percentage distribution socioeconomic and background factors of the adolescents age 15–24 in India, Bangladesh, Pakistan and Nepal. Majority of the adolescents belonged to the age group 20–24 in all the four countries. Majority of the adolescents in India, Bangladesh and Nepal were having secondary education while almost 50% of the adolescents in Pakistan were illiterate. In India and Nepal, majority of the adolescents were from Hindu religion while it was Muslims in case of Bangladesh. Less than 50% of the adolescents belonged to the advantaged section (rich) of the society in all the four countries. Majority of the female adolescents were employed in all the four countries. In case of India, majority of the adolescents initiated their first sexual activity at age 18 or above (59.19%) while in case of Bangladesh and Nepal, majority belonged to the age group 15–17. In all the four countries, majority of the adolescents were exposed to media. Majority of the adolescents in Bangladesh, Pakistan and Nepal had not visited by family planning workers.

**Table 1:** Sample distribution of currently married female adolescents in selected south Asian countries by various background characteristics, DHS

Background characteristics	India	Bangladesh	Pakistan	Nepal
*Age (years)*				
15–19	17,606 (18.72)	1954 (38.52)	576 (22.44)	730 (29.51)
20–24	76,427 (81.28)	3119 (61.48)	1992 (77.56)	1743 (70.49)
*Place of residence*				
Urban	24,364 (25.91)	1382 (27.24)	670 (26.10)	1358 (54.91)
Rural	69,669 (74.09)	3691 (72.76)	1898 (73.90)	1115 (45.09)
*Education*				
No education	16,654 (17.71)	383 (7.55)	1277 (49.73)	410 (16.59)
Primary	12,356 (13.14)	1353 (26.68)	523 (20.38)	502 (20.32)
Secondary	55,210 (58.71)	2778 (54.76)	573 (22.29)	1218 (49.25)
Higher	9813 (10.44)	558 (11.01)	195 (7.60)	342 (13.84)
*Religion*				
Hindu	75,802 (80.61)	334 (6.60)	NA	2121 (85.75)
Muslim	1356 (1.44)	4665 (91.95)	NA	158 (6.40)
Others	16,874 (17.95)	73 (1.45)	NA	194 (7.85)
*Wealth index*				
Poorest	19,522 (20.76)	970 (19.13)	596 (23.24)	455 (18.43)
Poorer	22,420 (23.84)	950 (18.87)	560 (21.79)	544 (22.02)
Middle	21,377 (22.73)	1013 (19.97)	541 (21.06)	609 (24.63)
Richer	18,524 (19.70)	1133 (22.34)	524 (20.39)	567 (22.92)
Richest	12,188 (12.96)	998 (19.69)	347 (13.52)	296 (11.99)
*Working status*				
No	13,940 (88.16)	3966 (78.21)	2071 (80.63)	1363 (55.13)
Yes	1872 (11.84)	1105 (21.79)	497 (19.37)	1110 (44.87)
*Age at first sex (years)*				
≥ 14	6203 (6.64)	1429 (28.18)	NA	241 (9.74)
15–17	31,932 (34.17)	2383 (46.97)	NA	1264 (51.11)
≤ 18	55,320 (59.19)	1261 (24.85)	NA	968 (39.14)
*No. of living children*				
0	32,508 (34.57)	1373 (27.07)	928 (36.16)	780 (31.56)
1	36,944 39.29)	2527 (49.80)	839 (32.67)	1138 (46.01)
2	20,328 (21.62)	1008 (19.87)	562 (21.88)	448 (18.12)
3 and above	4252 (4.52)	165 (3.26)	238 (9.29)	107 (4.31)
*Husband's education*				
No education	1878 (11.88)	848 (16.73)	762 (29.68)	220 (8.89)
Primary	2041 (12.91)	1601 (31.58)	530 (20.66)	478 (19.34)
Secondary	9468 (59.88)	1979 (39.01)	913 (35.55)	1261 (50.98)
Higher	2423 (15.33)	643 (12.69)	362 (14.11)	514 (20.79)
*Media exposure*				
No	19,785 (21.04)	1614 (31.85)	693 (27.00)	420 (16.99)
Yes	74,248 (78.96)	3455 (68.15)	1875 (73.00)	2053 (83.01)
*Visited by FP worker*				
No	NA	3991 (78.66)	851 (54.42)	1360 (54.97)
yes	NA	1082 (21.34)	712 (45.58)	1113 (45.03)
Total	94,034	5074	2569	2474

DHS = Demographic Health Survey; NA = Not Available; FP = Family Planning

#### Differentials in contraceptive use

Table 2 shows the percentage of contraceptive use among adolescents 15–24 by important socio-economic characteristics in selected countries. In all the four countries, adolescents in age group 20–24 were using more contraception than 15–19; the highest significant difference between the two age groups was observed in India. In all the four south Asian countries, the higher contraceptive use was found among women from urban backdrop as compared to their rural counterparts. The lowest level of contraceptive use has been found among illiterate adolescents; the level increased as the education of adolescents’ increase. In India, the highest level of contraception was found among Muslims, while in Nepal it was in Others, in Bangladesh it was in Hindus, while the data for religion was not taken in Pakistan. The lowest contraceptive use prevailed among adolescents from poorest wealth quintile, the level of which increased as the wealth index of the adolescents’ increase. Age of the adolescents at first sex showed significant relationship with their contraceptive use, except for Pakistan where the data about the same was not available. A major difference in contraceptive use was found owing to the employment of the adolescents. The ones who were working were using more contraception than those who did not work; except for Pakistan, every country had this similar pattern. In all the four countries, the adolescents having no children were using contraceptive much lesser than those having one, two, three or more children. In almost every country taken for analysis, contraceptive use was observed low among women having illiterate husband whereas it was high among women who were exposed to media than their counterparts. Lastly, in Bangladesh, Nepal and Pakistan, the women who were visited by FP worker in last 12 months had higher contraceptive use than those who were not exposed to visits by FP worker.

**Table 2:** Bivariate association of currently married female adolescents and contraceptive use in selected south Asian countries by various background characteristics, DHS

Background characteristics	India	Bangladesh	Pakistan	Nepal
*Age (years)*				
15–19	9.96*	46.74*	6.89*	14.48*
20–24	23.56*	54.55*	14.88*	23.95*
*Place of residence*				
Urban	24.13*	57.20*	18.15*	22.93*
Rural	19.92*	49.42*	11.30*	18.99*
*Education*				
No education	16.06*	50.68	8.55*	15.93
Primary	23.08*	52.19	15.98*	20.42
Secondary	22.50*	51.07	19.80*	22.67
Higher	18.43*	52.89	15.31*	23.11
*Religion*				
Hindu	20.69*	56.05	NA	21.28*
Muslim	23.31*	51.29	NA	7.23*
Others	22.28*	46.93	NA	31.11*
*Wealth index*				
Poorest	15.39*	50.20	6.68*	22.53
Poorer	20.47*	51.55	10.73*	22.79
Middle	22.28*	51.78	13.89*	18.72
Richer	24.06*	52.05	17.59*	19.65
Richest	24.16*	52.01	19.85*	23.90
*Working status*				
No	21.03*	48.99*	13.43	17.73*
Yes	26.93*	60.57*	11.67	25.36*
*Age at first sex (years)*				
≥ 14	32.22*	58.30*	NA	28.70*
15–17	25.13*	50.27*	NA	19.77*
≤ 18	17.45*	51.54*	NA	21.08*
*No. of living children*				
0	5.51*	25.11*	0.53*	8.49*
1	20.66*	59.46*	14.59*	23.78*
2	41.96*	66.40*	25.27*	31.59*
3 and above	42.40*	59.31*	27.99*	41.99*
*Husband's education*				
No education	18.87*	53.54	10.85*	15.49
Primary	26.62*	52.17	11.48*	22.52
Secondary	21.78*	49.68	12.90*	21.23
Higher	19.65*	53.15	20.63*	22.12
*Media Exposure*				
No	13.66*	48.42*	5.99*	14.07*
Yes	22.97*	53.04*	15.71*	22.60*
*Visited by FP worker*				
No	NA	49.82*	11.53*	17.54*
Yes	NA	57.87*	20.33*	25.57*

DHS = Demographic Health Survey; NA = Not Available; FP = Family Planning

*Significant (p < 0.05)

#### Determinants of contraceptive use among female adolescents

Table 3 shows the odds from logistic regression model done for all the four countries taken for the analysis. The logistic regression recognised education, religion, wealth, work status, age at first sex, number of living children, media exposure, visit by FP worker as significant predictors affecting the contraceptive use among female adolescents. Here, age and place of residence lost the significance as predictors affecting the contraceptive use. Higher education was associated with a drastic [India: 1.37*(1.06, 1.57), p = 0.005; Bangladesh- 1.85*(1.36, 2.51), p = 0.005; Pakistan- 2.3*(1.54, 3.43), p = 0.005; Nepal- 1.42(0.92, 2.2), p = 0.052] increase in the contraception use among female adolescents in all four countries. Female adolescents who belonged to Muslim [India-0.54*(0.43, 0.68); Bangladesh, 0.93 (0.74, 1.17) and Nepal, 0.31*(0.16, 0.62)] and other religion were having lower likelihood of using contraception in all countries except for Pakistan where the data on religion was not available. Wealth did not appear as a significant predictor of contraceptive use in Bangladesh and Nepal. Whereas, in India and Nepal, being wealthy was associated with manifold increase in contraceptive use [India: 2.41*(2, 2.9) and Nepal: 1.54*(1.02, 2.34)]. Adolescents who were currently working were significantly more likely to use contraception in India (1.24*(1.09, 1.41)), Bangladesh (1.63*(1.39, 1.9)) and Nepal (1.36*(1.1, 1.68)). Those adolescents who have initiated the sexual union after age 15–17 were significantly associated with reduction of contraceptive use in India (0.73*(0.61, 0.86)) Nepal (0.68*(0.49, 0.95)) when compared to the younger age group (≥ 14). Having one or more than one children was associated with manifold increase in contraceptive use than those who do not have any child [(India: 10.27*(8.84, 11.92)], Bangladesh: 8.07*(6.48, 10.06), Pakistan: 66.77*(20.73, 215.09), Nepal: 6.68*(4.6, 9.69)). Being exposed to media (radio, television and newspaper) was related to significant increase in contraceptive use among adolescents in all the four countries (66% (1.45, 1.89), 24% (1.07, 1.45), 68% (1.1, 2.55) and 68% (1.2, 2.37) in India, Bangladesh, Pakistan and Nepal respectively). Similarly, visit by FP worker in last 12 months was significantly associated (Bangladesh: 1.26*(1.09, 1.47), Pakistan 1.33*(1, 1.78), and Nepal 1.26*(1.02, 1.55)) with increase in contraceptive use in all countries in the analysis.

**Table 3:** Odds ratio of currently married female adolescents using contraceptives in selected south Asian countries by various background characteristics, DHS

Background characteristics	India	Bangladesh	Pakistan	Nepal
*Age (years)*				
15–19®	1	1	1	1
20–24	1.03 (0.89, 1.19)	0.69* (0.6, 0.8)	1.01 (0.63, 1.61)	0.96 (0.72, 1.27)
*Place of residence*				
Urban®	1	1	1	1
Rural	1 (0.9, 1.11)	0.69* (0.6, 0.79)	0.82 (0.6, 1.12)	0.81 (0.65, 1.01)
*Education*				
No education®	1	1	1	1
Primary	1.29* (1.17, 1.6)	1.16 (0.9, 1.49)	1.75* (1.15, 2.65)	1.21 (0.82, 1.76)
Secondary	1.30* (1.14, 1.48)	1.25 (0.97, 1.6)	2.3* (1.54, 3.43)	1.40 (0.98, 2)
Higher	1.37* (1.06, 1.57)	1.85* (1.36, 2.51)	1.86* (1.08, 3.2)	1.42 (0.92, 2.2)
*Religion*				
Hindu®	1	1	NA	1
Muslim	0.54* (0.43, 0.68)	0.93 (0.74, 1.17)	NA	0.31* (0.16, 0.62)
Others	0.98 (0.87, 1.09)	0.79 (0.35, 1.77)	NA	1.42 (1, 2.01)
*Wealth index*				
Poorest®	1	1	1	1
Poorer	1.32* (1.14, 1.52)	1.07 (0.88, 1.3)	0.97 (0.57, 1.68)	1.25 (0.92, 1.69)
Middle	1.54* (1.33, 1.79)	1 (0.81, 1.23)	1.09 (0.63, 1.89)	1.23 (0.9, 1.69)
Richer	1.79* (1.52, 2.11)	0.97 (0.78, 1.2)	1.06 (0.59, 1.9)	1.16 (0.82, 1.62)
Richest	2.41* (2, 2.9)	0.85 (0.67, 1.08)	1.05 (0.55, 2.03)	1.54* (1.02, 2.34)
*Working status*				
No®	1	1	1	1
Yes	1.24* (1.09, 1.41)	1.63* (1.39, 1.9)	0.87 (0.54, 1.4)	1.36* (1.1, 1.68)
*Age at first sex (years)*				
≥ 14®	1	1	NA	1
15–17	0.73* (0.61, 0.86)	0.91 (0.78, 1.05)	NA	0.68* (0.49, 0.95)
≤ 18	0.62* (0.52, 0.73)	0.9 (0.75, 1.09)	NA	0.85 (0.58, 1.25)
*No. of living children*				
0®	1	1	1	1
1	4.05* (3.54, 4.63)	5.24* (4.47, 6.15)	40.63* (12.77, 129.25)	3.53* (2.62, 4.76)
2	10.27* (8.84, 11.92)	8.07* (6.48, 10.06)	66.77* (20.73, 215.09)	6.68* (4.6, 9.69)
3 and above	12.03* (9.7, 14.91)	4.9* (3.38, 7.1)	98.11* (29.53, 325.95)	12.08* (6.93, 21.06)
*Media exposure*				
No®	1	1	1	1
Yes	1.66* (1.45, 1.89)	1.24* (1.07, 1.45)	1.68* (1.1, 2.55)	1.68* (1.2, 2.37)
*Visited by FP worker*				
No®	NA	1	1	1
Yes	NA	1.26* (1.09, 1.47)	1.33* (1, 1.78)	1.26* (1.02, 1.55)

DHS = Demographic Health Survey; NA = Not Available; FP = Family Planning

*Significant (p < 0.05)

®Reference category

Table 4 shows the results of concentration index for contraceptive use in India, Bangladesh, Nepal and Pakistan with a bifurcation of rural and urban residence. The socioeconomic inequality for India was observed as 0.80, 0.06 for Bangladesh, − 0.013 for Nepal and 0.198 for Pakistan. For India, the regions depicting the negative values for concentration index were North- east (C.I. = − 0.017) and south (C.I. = − 0.054). Similar values came for rural as well urban areas in these regions as well. It showed that the health outcome (contraceptive use) is more concentrated among the poor population in these two regions. While all the regions of Bangladesh showed positive values of concentration index, the rural areas of Barisal (C.I. = − 0.032), Chittagong (C.I. = − 0.153) and Rangpur (C.I. = − 0.007) depicted inequality in contraceptive use with a disproportionate burden among poor population; in urban areas it was for the regions of Chittagong and Rajshahi. In case of Nepal, province 1 (C.I. = − 0.059) and province 5 (C.I. = − 0.045) showed the negative values in both rural and urban areas; while it was concentrated in rural (C.I. = − 0.068) and urban (C.I. = − 0.015) areas of province 2 and 4 respectively. In Pakistan, all the values were positive except for urban areas of Gilgit Baltistan (C.I. = − 0.062).

**Table 4:** Concentration index of modern contraceptive use among currently married female adolescents in regions of selected four south Asian countries, DHS

	Total		Rural		Urban	
	CI	SE	CI	SE	CI	SE
*INDIA*	*0.80*					
North	0.133	0.007	0.137	0.009	0.124	0.015
central	0.151	0.008	0.121	0.009	0.113	0.014
East	0.135	0.007	0.153	0.008	0.026	0.015
North-east	− 0.017	0.008	− 0.015	0.009	0.019	0.020
West	0.045	0.012	0.015	0.014	0.075	0.021
South	− 0.054	0.010	− 0.034	0.012	− 0.112	0.018
*BANGLADESH*	*0.06*					
Barisal	0.238	0.021	− 0.032	0.025	0.074	0.039
Chittagong	0.186	0.023	− 0.153	0.031	− 0.030	0.030
Dhaka	0.195	0.018	0.066	0.025	0.006	0.024
Khulna	0.192	0.018	0.007	0.023	0.001	0.032
Rajshahi	0.217	0.017	0.002	0.020	− 0.050	0.029
Rangpur	0.223	0.016	− 0.007	0.019	0.003	0.032
Sylhet	0.297	0.035	0.120	0.044	0.125	0.055
*NEPAL*	− *0.013*					
Province 1	− 0.059	0.051	− 0.048	0.084	− 0.057	0.065
Province 2	0.058	0.073	− 0.068	0.117	0.218	0.093
Province 3	0.005	0.061	0.100	0.099	− 0.0312	0.0786
Province 4	0.069	0.062	0.185	0.120	− 0.015	0.072
Province 5	− 0.045	0.053	− 0.007	0.092	− 0.108	0.064
Province 6	0.034	0.055	0.025	0.086	0.186	0.072
Province 7	0.136	0.055	0.332	0.080	0.009	0.074
*PAKISTAN*	*0.198*					
Punjab	0.082	0.052	0.028	0.066	0.174	0.082
Sindh	0.412	0.069	0.482	0.153	0.034	0.072
Khyber Pakhtunkhwa	0.264	0.063	0.302	0.080	0.040	0.092
Balochistan	0.194	0.111	0.191	0.155	0.115	0.138
Gilgit Baltistan	0.181	0.092	0.096	0.141	− 0.062	0.097
Islamabad	0.044	0.097	0.123	0.125	0.107	0.160

CI = Concentration Index, SE = Standard Error

#### Depicting inequality in contraceptive use through concentration curves

Aligning to the positive and negative values of concentration index, the concentration curve shows the inequality in distribution of the health outcome for the four countries taken up for the analysis. The concentration curve for all the four countries show the cumulative proportion of the variable against the cumulative proportion of currently married adolescent women in the age group 15–24 years. The concentration index is a 45° line known as the line of equality, if the health outcome, in our case contraceptive use, is equally distributed among the rich as well poor sections of the society. If the curve lies above the line of equality, it indicated the disproportionate burden of the outcome variable among the poor while its vice-versa if the line lies below the line of equality.

**Fig. 1:**
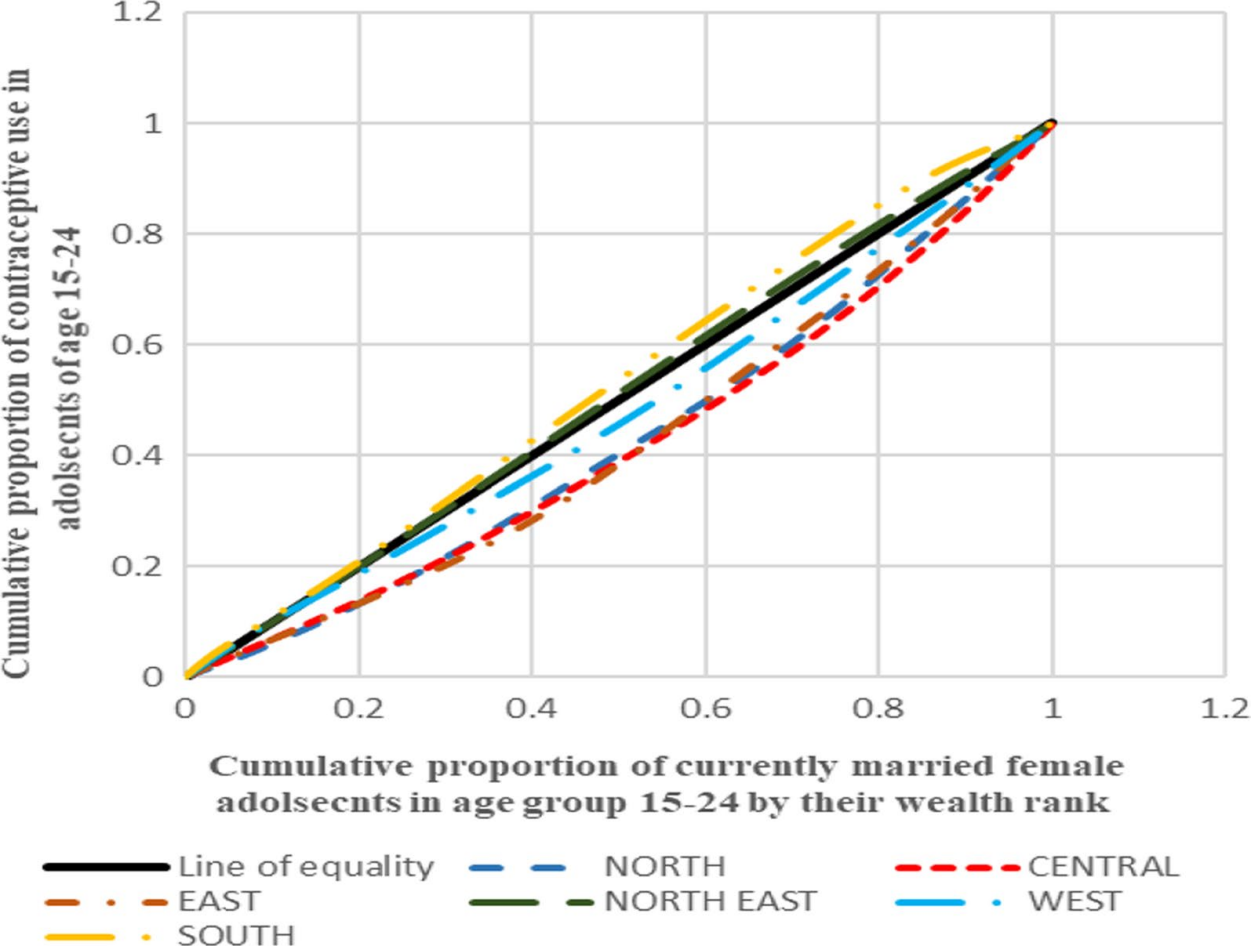
Concentration curves for inequality in contraceptive use among female adolescents in India, NFHS 2015-16

**Fig. 2:**
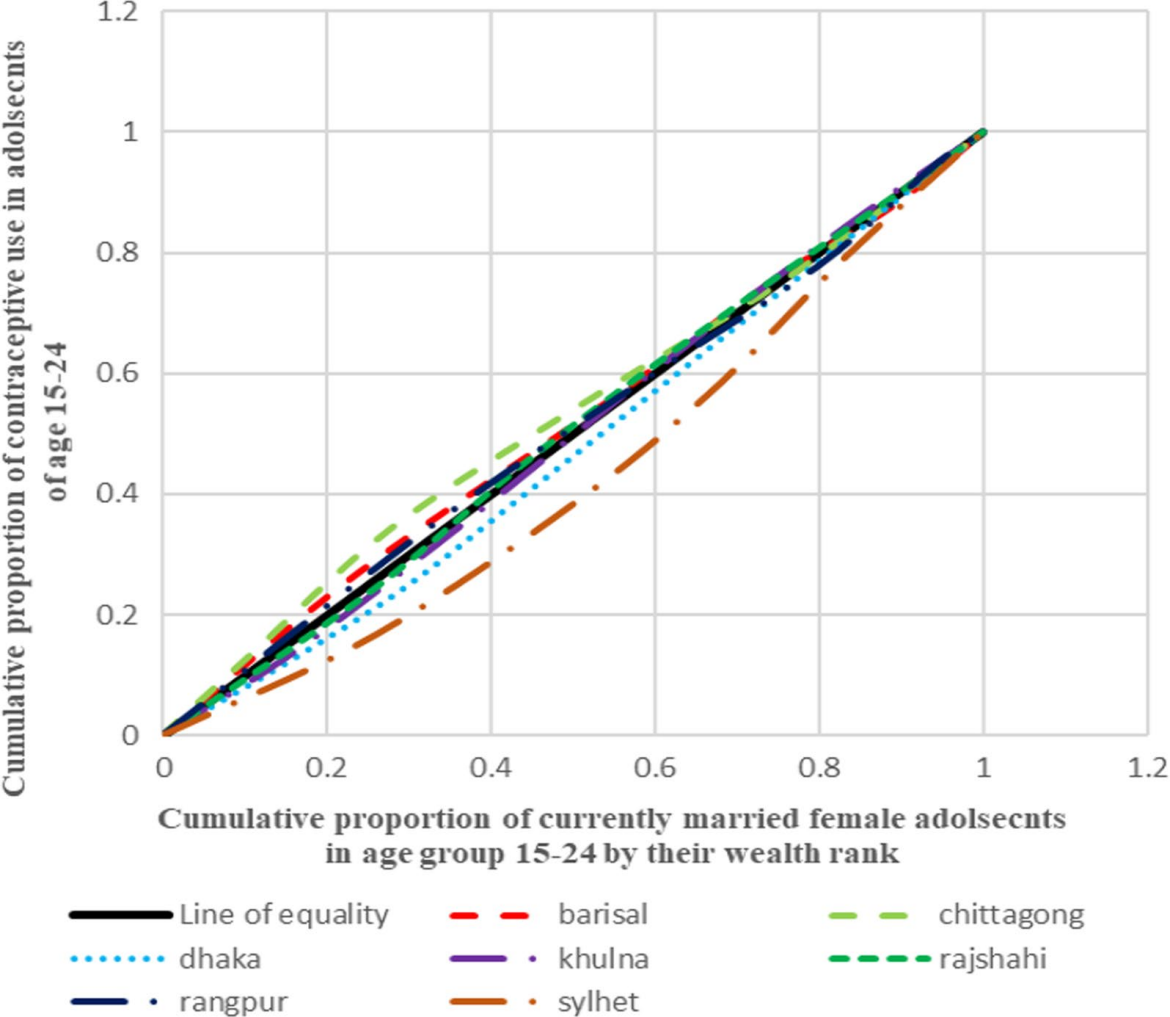
Concentration curves for inequality in contraceptive use among female adolescents in Bangladesh, DHS 2014

**Fig. 3:**
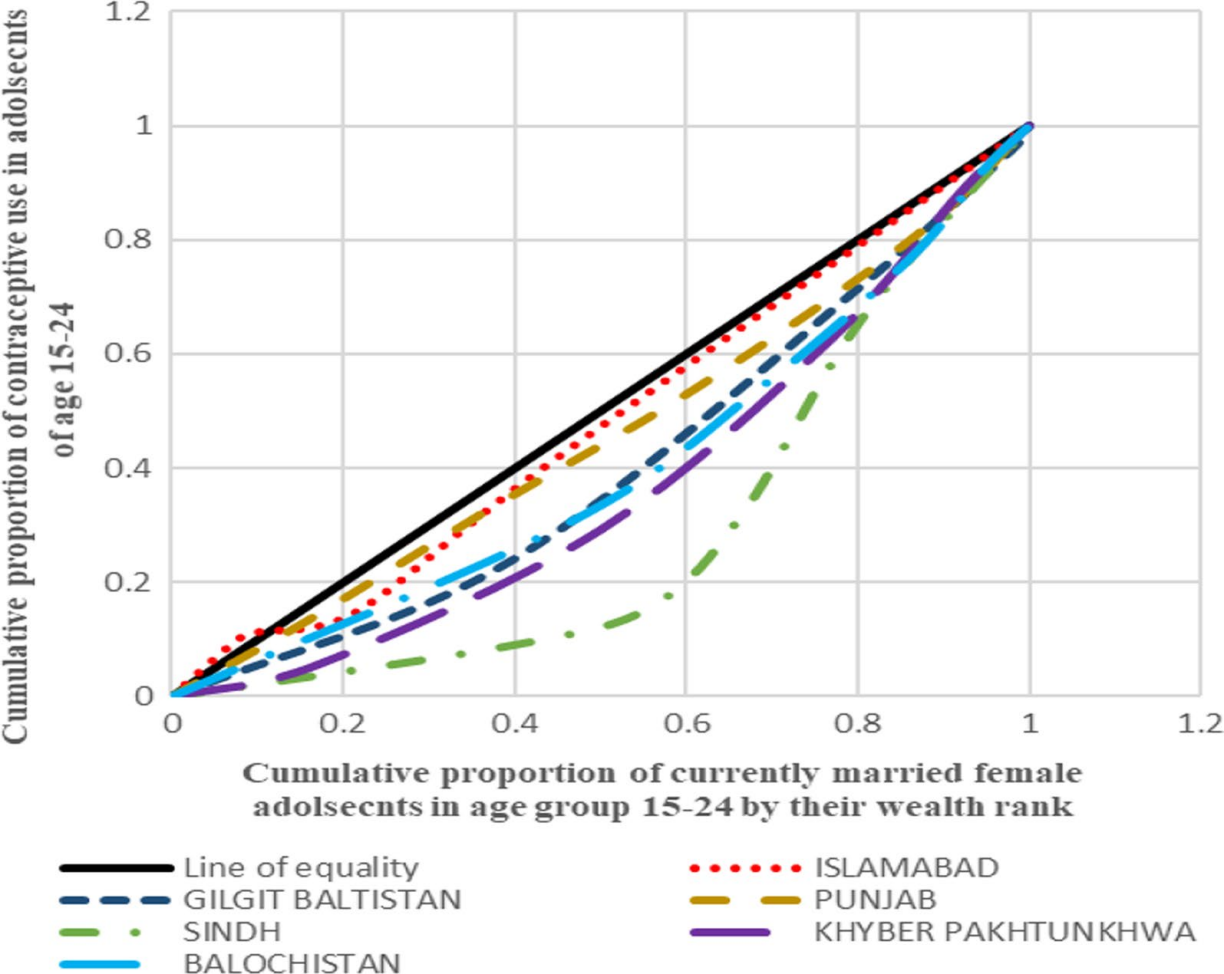
Concentration curves for inequality in contraceptive use among female adolescents in Pakistan, DHS 2012-13

**Fig. 4:**
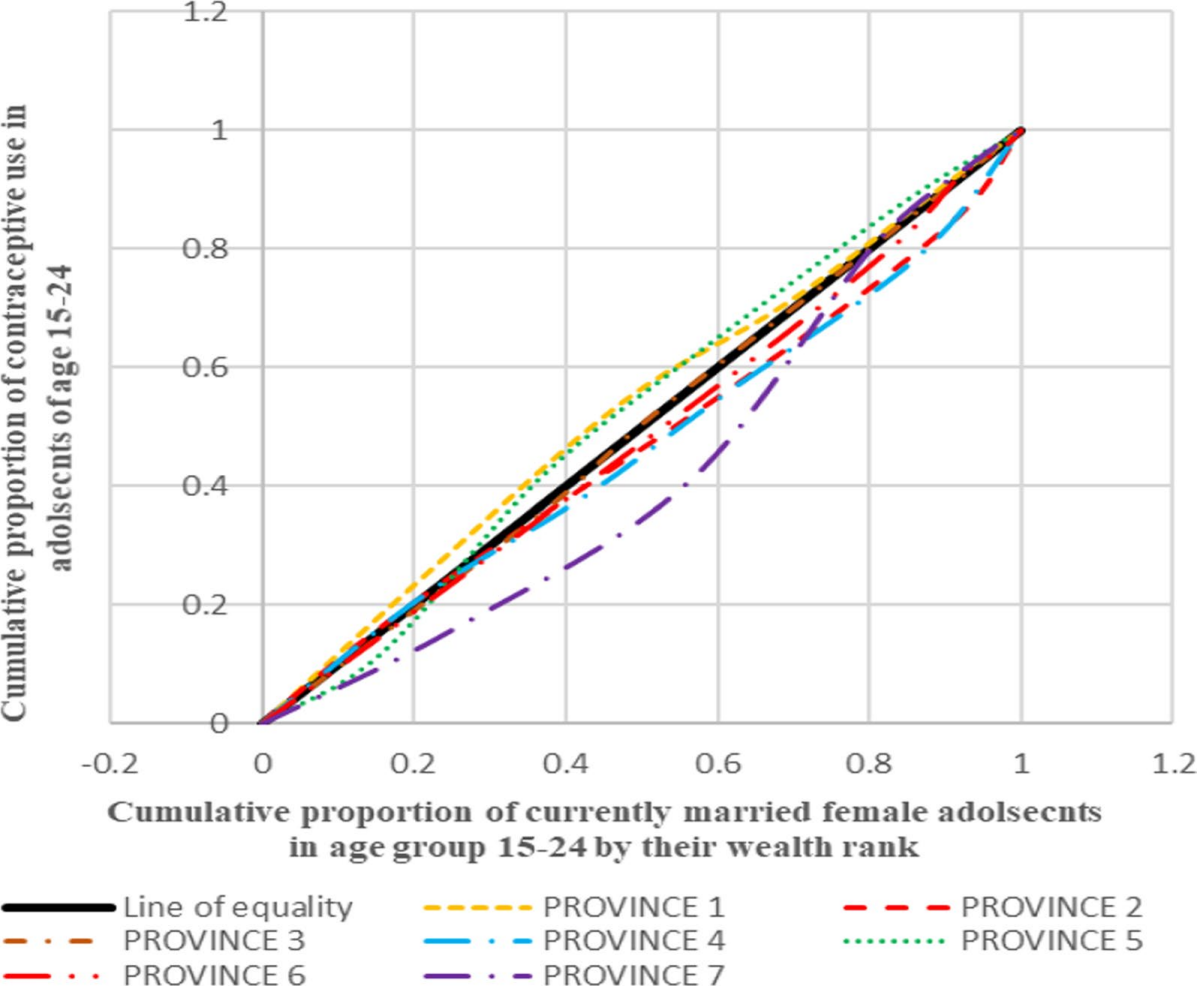
Concentration curves for inequality in contraceptive use among female adolescents in Nepal, DHS 2016

**Fig. 5:**
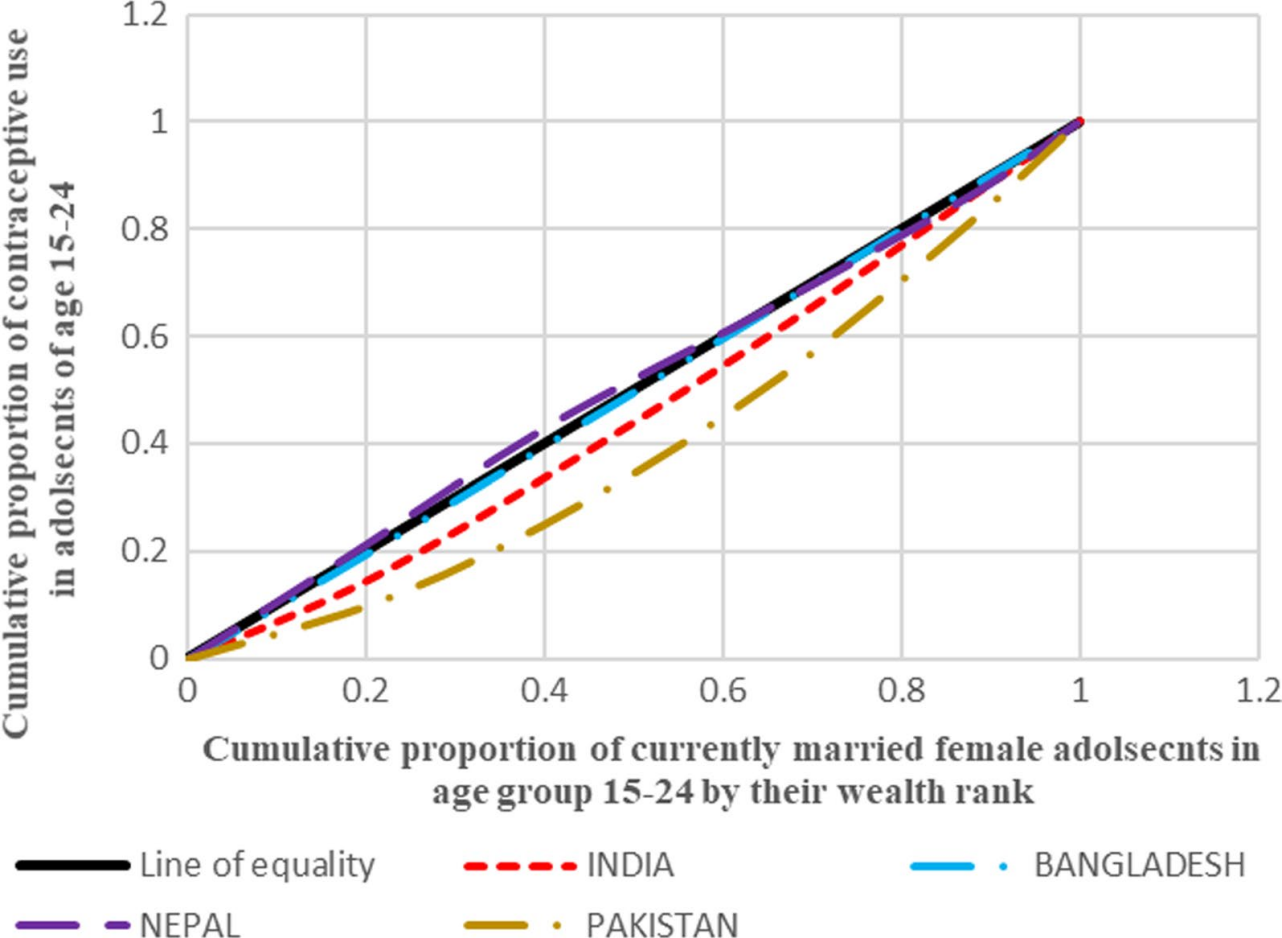
Concentration curves for inequality in contraceptive use among female adolescents in selected South Asian countries, DHS

Figure 1 presents the concentration curves plotted for regions of India which shows that the Eastern region was the most diverged from the line of equality followed by Central, North and West. In all these regions, contraceptive use was more concentrated among the advantaged section of the population. On contrary to this, Northeast and Southern regions lied above the line of equality which showed the concentration of the outcome in the richest lot. Figure 2 shows the concentration curves made for the regions of Bangladesh. It was found that Sylhet was the most diverged region from the line of equality followed by Dhaka (capital), Rangpur and Rajshahi. The curves going below the line of equality in all these four regions depicted that the advantaged section of the population had an upper hand when it comes to contraceptive use. The regions of Khulna, Barisal and Chittagong lied above the line of equality showing the preponderance of contraceptive use among the poor adolescents. Figure 3, in Nepal, four (Province 2, 4, 6 and 7) provinces were found to be lying below the line of equality, in which Province 7 showed the maximum diversion. Province 1 and 5 were above the line of equality which represents a scenario where contraceptive use is concentrated amongst the poor individuals. The curve for Province 3 was almost similar to the line equality depicting the equal contraceptive use in both advantaged and disadvantaged section of the population. Figure 4, in Pakistan, every region lies below the line of equality which represents a disappointing situation where the use of contraception is highly concentrated among the advantaged section of the society. The maximum diversion was seen in Sindh, followed by Khyber Pakhtunwala, Balochistan, Gilgit Baltistan. A somehow better situation is found in the capital Islamabad in which the curve lies near the line of equality depicting a better situation compared to the rest. Figure 5, the results of the analysis showed that the curves plotted for Pakistan was most diverged from the line of equality, followed by India. The curves were below the line of equity which meant that use of contraceptive was concentrated among the richest population, which depicts the condition among the economically disadvantaged group. The concentration curve for Bangladesh lied just upon the line of equality depicting the use of contraception as fair and equally distributed among the rich as well as the poor adolescents. However, Nepal showed the curve which was a bit above the line of equality which represents a situation where contraceptive use was more concentrated among the poor population.

#### Decomposing the socioeconomic inequality in contraceptive use

Table 5 presents the results of the decomposition analysis carried out in order to determine the contribution various socio-economic and demographic factors in the inequality in contraceptive use among the adolescent population. The results revealed that the socio-economic and demographic factors played an important part in explaining the inherent inequality in contraceptive use among Asian adolescents. In case of India, poor economic status (95.23% to the explained part of inequalities), illiteracy of the respondents (51.29%), and rural place of residence (23.06%) contributed maximum in explaining the inequality in contraceptive use among female adolescents. The highest elasticities were observed with respect to age (0.141) and illiteracy (0.069). For Bangladesh, the largest contributors to inequalities in contraception were rural place of residence (260%), illiteracy (146.67%) while birth order 3 + (− 173.33%) contributed negatively to existing inequality in contraceptive use. Alike India, the highest elasticities were found with respect to illiteracy (0.057) and age (0.046). Illiteracy (50%), poor economic status (47.83%) and rural place of residence (16.30%) contributed maximum to the inequalities in contraceptive use among adolescents in Pakistan while birth order 3 + (− 9.78%) contributed negatively. Highest elasticities were observed with birth order 3 + (0.060) and illiteracy (0.036). In Nepal, the important operators of inequalities in contraceptive use among female adolescents were unemployment (105.26%), birth order 3 + (52.63%) and poor economic status (47.37%), while rural place of residence contributed negatively (− 63.16%) to inequalities in contraceptive use. Overall, India and Bangladesh displayed a similar pattern where age, rural place of residence and illiteracy were significantly the highest contributors in inequalities in contraception. In case of Pakistan and Nepal, it was rural place of residence and poor economic status which contributed most to the inequalities while birth order 3 + contributed negatively to inequality in all the four countries.

**Table 5:** Effect and contribution of predictor variables of modern contraceptive use among female adolescents based on decomposition analysis in selected south Asian countries

Predictors	Elasticity	CI	Absolute	% contribution
*INDIA, N* = *94,034*				
20–24 age (years)	0.1418	0.0149	0.0084	16.70
Rural place of residence	− 0.0446	− 0.0648	0.0116	23.06
Poor economic status	0.0268	0.446	0.0479	95.23
Illiterate	0.0696	0.0926	0.0258	51.29
Unemployed	NA	NA	NA	NA
Illiterate partner	NA	NA	NA	NA
Vulnerable age at first sex (years)	− 0.0392	0.1183	− 0.0186	− 36.98
Birth order 3 +	0.0687	− 0.0903	− 0.0248	− 49.30
Total			**0.0503**	**100.00**
*BANGLADESH, N* = *5074*				
20–24 age (years)	0.046	0.009	0.001	6.67
Rural place of residence	− 0.154	− 0.064	0.039	260.00
Poor economic status	− 0.001	0.26	− 0.001	− 6.67
Illiterate	0.057	0.098	0.022	146.67
Unemployed	0.021	− 0.088	− 0.007	− 46.67
Illiterate partner	− 0.01	0.072	− 0.002	− 13.33
Vulnerable age at first sex (years)	− 0.015	0.18	− 0.011	− 73.33
Birth order 3 +	0.039	− 0.168	− 0.026	− 173.33
Total			**0.015**	**100**
*PAKISTAN, N* = *2569*				
20–24 age (years)	0.01	0.013	0.0	0.00
Rural place of residence	− 0.046	− 0.081	0.015	16.30
Poor economic status	0.024	0.45	0.044	47.83
Illiterate	0.036	0.32	0.046	50.00
Unemployed	0.003	− 0.357	− 0.004	− 4.35
Illiterate partner	− 0.001	0.137	0	0.00
Vulnerable age at first sex (years)	NA	NA	NA	NA
Birth order 3 +	0.06	− 0.038	− 0.009	− 9.78
Total			**0.092**	**100**
*NEPAL, N* = *2474*				
20–24 age (years)	0.082	0.014	0.004	− 21.05
Rural place of residence	− 0.061	− 0.049	0.012	− 63.16
Poor economic status	− 0.005	0.404	− 0.009	47.37
Illiterate	0.06	0.009	0.002	− 10.53
Unemployed	0.029	− 0.175	− 0.02	105.26
Illiterate partner	0.04	0.011	0.001	− 5.26
Vulnerable age at first sex (years)	0.002	0.094	0.001	− 5.26
Birth order 3 +	0.038	− 0.07	− 0.01	52.63
Total			− **0.019**	**100.00**

Totals of every country are provided in bold

DHS = Demographic Health Survey; C.I. = Concentration Index; N = Sample; NA = Not Available

## Discussion and Conclusion

The study is dedicated to explore the important predictors of contraceptive use and decompose the socioeconomic inequalities in contraceptive use among female adolescents in the sample of four Asian countries. Although the knowledge of contraception is high in these countries, but the use of contraception remains low. A higher level of knowledge about contraception does not always translate into a higher level of contraceptive use [7]. A recent study in Pakistan sheds light on this fact that despite 93.4% of women having knowledge of contraception, only 49.7% currently used contraception [15].

The findings of the study reveals that age and contraceptive use were not significantly associated with each other, this finding was found in accordance with a similar study as well [16]. In the bivariate analysis, contraceptive use was highly and significantly associated with the place of residence, wherein adolescents living in the urban areas showed higher preponderance of contraceptive use in all the four countries. However, this association became insignificant when other factors were controlled in the regression analysis. Similar findings were provided by a study on Bangladeshi women in which women from rural setting had less chance of contraceptive use than a woman from urban setting [17].

Socioeconomic factors played an important role in contraceptive usage among adolescents. Education and media exposure showed significant positive relationship with contraceptive use in all the four countries as well. Adolescents who were educated and aware of contraception and its benefits through media exposure used more contraceptive than their respective counterparts [18]. Additionally, the study revealed that wealthier women were more likely to use contraceptives than poor women. This might be due the capacity to purchase modern contraceptives not necessarily relying on their partners [19]. It was found that contraceptive use was more among women with two or more children compared to women with no children. A study based on data from 73 lower-middle income countries (LMIs) revealed a similar finding where modern contraceptive prevalence in most countries were particularly low among married adolescents without children in comparison to those having children [20]. Contraceptive use was more predominantly found among adolescents who were visited by FP worker in last 12 months, this finding is tune with a previous study which anticipated that students with more frequent contacts with the family planning program and among those who received family planning services in combination with other medical or counselling services [9]. A critical question is whether method choice is pre-dominantly supply driven or demand driven. The question that e women are using the particular contraceptive methods that they prefer to use because the providers recommend them or because they are available at local facilities is a matter of concern [21].

The study further assessed the socioeconomic inequalities in contraceptive use among female adolescents in the selected countries. A significant amount of variation has been observed across the countries. Pakistan showed the greatest socio-economic inequality in contraceptive use, followed by India. while Nepal showed a better picture with lesser socio-economic inequality, Bangladesh depicted no socio-economic inequality in contraceptive use. Among all the socio-economic factors taken in the analysis, the major contributing factors to socio-economic inequality in contraceptive use vary by country. In case of India and Bangladesh, age, illiteracy and birth order 3 + contributed most to the inequality. Somehow India and Bangladesh depicted a similar pattern, whereas, in Pakistan, it is illiteracy, age and birth order 3 + and poor economic status. Age, illiteracy, illiterate partner are the factors that contribute the bulk of inequalities in contraceptive use in Nepal. This proves social and economic constraints in accessing services and inadequacies in health care system is still a major issue of concern in these Asian countries [8].

Using a cross country perspective, this study captured the inequality in contraceptive use in India, Nepal, Pakistan and Bangladesh. Factors like education, employment, having one or more children, media exposure were positively associated with contraceptive use among adolescents in all the four countries taken for the analysis. Cross-national research based on impact of community level factors among youth in low and middle income countries are few, especially on contraceptive use, which are necessary for the grass root level interventions for family planning use [22].

Female adolescents represent a segment of the population which is less aware than others about the sexual and reproductive matters, less experienced as they have just entered this phase of transition. While this ought to be problems from the demand side, there are problems which remain there by the supply side as well. Availability, accessibility to contraceptives is an important issue to be taken into consideration while framing a policy in this context. The policy initiative should be focused towards socio-economic factors such as poor economic status, illiteracy, have more children etc. to reduce the inherent inequalities in contraceptive use among adolescents. Since the factors contributing to inequalities in contraceptive use vary across countries, there is a need to imply country-specific initiatives which will look after the special needs of this adolescents age-group. The small scale interventions will definitely help to achieve equity in contraceptive use among female adolescents in these countries.

## Data Availability

The data is available in the public domain and can be accessed from the official site of The DHS Program: Demographic and Health Surveys (https://dhsprogram.com). The following link can be trailed for directly assessing the data sets section and by requesting permission on https://dhsprogram.com/data/available-datasets.cfm. Moreover, the datasets used and/or analysed during the current study can be made available from the corresponding author on reasonable request.
